# Anthropometric and Physical Performance Reference Values in Young Handball Players Aged 9–15 Years: A Cross-Sectional Study Using Percentile Profiling and Factorial ANOVA

**DOI:** 10.3390/jfmk11030250

**Published:** 2026-06-26

**Authors:** Samir Krichen, Chirine Aouichaoui, Hamada Chaari, Liwa Masmoudi, Yousri Elghoul, Monia Zaouali, Wajdi Dardouri, Hamdi Chtourou, Yassine Trabelsi, Mohamed Zouch

**Affiliations:** 1 Research Laboratory of Exercise Physiology and Pathophysiology: From Integral to Molecular “Biology, Medicine and Health” (LR19ES09), Faculty of Medicine of Sousse, University of Sousse, Sousse 4000, Tunisia; samirkrichen2@yahoo.fr (S.K.); chirineaouichaoui@yahoo.com (C.A.); chaarihamada@yahoo.fr (H.C.); zaoualimonia@yahoo.fr (M.Z.); trabelsiyassine@yahoo.fr (Y.T.); 2 High Institute of Sport and Physical Education of Ksar Said, University of Manouba, Manouba 2010, Tunisia; 3 Research Laboratory: Education, Motricity, Sport and Health, EM2S, LR19JS01, High Institute of Sport and Physical Education of Sfax, University of Sfax, Sfax 3029, Tunisia; liwa.masmoudi@yahoo.fr (L.M.); yosri.elghoul@isseps.usf.tn (Y.E.); 4 Department of Sport Sciences and Physical Activity, College of Education, University of H’ail, Hail 81422, Saudi Arabia; wajdi.dardouri@gmail.com; 5 Physical Activity, Sport, and Health, UR18JS01, National Observatory of Sport, Tunis 1003, Tunisia; hamdi.chtourou@isseps.usf.tn

**Keywords:** physical fitness, sports development, exercise, body composition

## Abstract

**Background**: Reference values may assist practitioners in interpreting anthropometric and physical performance profiles in youth handball players within comparable sporting contexts. This study aimed to establish sex- and competitive-age-specific anthropometric and physical performance reference values for Tunisian youth handball players aged 9–15 years and to examine differences by sex and competitive age category. **Methods**: A total of 370 competitive youth handball players participated in this cross-sectional study (182 boys and 188 girls; U11, n = 130; U13, n = 158; U15, n = 82). Participants had at least two years of structured handball training. Assessment included body size, body composition, flexibility, squat jump, countermovement jump, 3 kg medicine ball throw, horizontal jumps, and handgrip strength. Sex, competitive age category, and sex × age category effects were examined using two-way ANOVA, with Bonferroni-adjusted post-hoc comparisons applied when appropriate. Effect sizes were reported as partial eta squared. Percentile values were calculated. Significance was set at *p* < 0.05. **Results**: Boys demonstrated higher values than girls in squat jump, (η_p_^2^ = 0.099), countermovement jump (η_p_^2^ = 0.097), medicine ball throw (η_p_^2^ = 0.202), and both dominant (η_p_^2^ = 0.073) and non-dominant handgrip strength (η_p_^2^ = 0.048, *p* < 0.001). Additionally, older age categories showed higher scores on all these tests (*p* < 0.001). Sex- and competitive-age-category-specific percentile values were established. **Conclusions**: The established reference values may support descriptive benchmarking and training/monitoring among comparable Tunisian youth handball players. However, these values should not be interpreted as maturity-adjusted standards or general population norms.

## 1. Introduction

Handball is an intermittent high-intensity team sport characterized by frequent accelerations, rapid changes of direction, jumps, high-velocity throws, body contacts, and rapid offensive and defensive transitions [1,2,3,4,5]. These match-play demands require the integration of aerobic and anaerobic energy systems, as well as neuromuscular qualities such as strength, power, coordination, braking capacity, and efficient force transfer [1,3,4,5]. The assessment of anthropometric and physical performance characteristics is particularly important because performance capacities vary across chronological age categories and between sexes due to growth, training exposure, motor learning, and inter-individual developmental variability [6,7,8].

Handball-specific performance relies on several interacting physical qualities from a physiological and biomechanical perspective. Jumping and horizontal propulsion require rapid lower-limb force production and effective use of the stretch–shortening cycle, whereas throwing actions require coordinated force transfer from the lower limbs and trunk to the upper limbs [9,10,11]. Handgrip strength may also contribute to ball control, throwing stability, and contact situations [12,13,14,15]. Previous training studies and reviews in youth handball and youth athletes indicate that strength, sprinting, jumping, and throwing capacities can be improved through appropriately designed training programs [16,17,18]. However, the present investigation is not an intervention study; therefore, these mechanisms are used only to justify the relevance of the selected field tests and not to infer training-induced adaptation.

The interpretation of anthropometric and physical performance characteristics in young athletes is complicated by growth, biological maturation, and relative age effects [19,20,21,22,23,24]. Players of the same chronological age may differ considerably in biological maturity, leading to large variability in strength, power, coordination, body mass, and fat-free mass [19,20,21,24]. This variability can bias early talent identification, favoring early-maturing individuals who may temporarily outperform their later-maturing peers [22]. Several studies in youth sport and youth handball found that maturity status and relative age may influence physical fitness, selection processes, and the interpretation of performance [21,22,23]. These factors are important because early physical advantages may reflect temporary growth or maturation differences rather than stable long-term athletic potential [25,26,27].

Few studies have investigated North African populations, where environmental, nutritional, and cultural factors may influence growth and performance trajectories [17,22]. Tunisia, a nation with a strong handball tradition, lacks comprehensive reference data for players across developmental stages. Recently, Aouichaoui et al. [28] provided reference values for Tunisian handball players aged 13–19 years while considering biological maturity status. However, limited information exists on younger Tunisian players aged 9–15 across competitive categories. Establishing reference values for these competitive group categories helps coaches, trainers, and sport scientists monitor physical profiles, assess strengths and weaknesses, and determine training priorities [25,27,28].

Accordingly, this cross-sectional study aimed to describe anthropometric, body composition, and physical performance characteristics of Tunisian youth handball players aged 9–15 years according to sex and competitive age category; to examine the effects of sex, age category, and their interaction on these outcomes; and to establish sex- and age-category-specific percentile reference values. We hypothesized that older competitive age categories would exhibit higher values in selected anthropometric and physical performance variables than younger categories, and that boys would exhibit higher values than girls in selected strength- and power-related outcomes.

## 2. Materials and Methods

### 2.1. Participants

This cross-sectional descriptive study was conducted among competitive youth handball players registered in regional training centers (Tunis-North, Sahel-East, Cap-Bon, Sfax-South) and clubs across Tunisia. The study sample comprised 378 players; only 370 were retained (182 boys and 188 girls), aged 9–15 years, who had at least two years of structured handball training. Participants were grouped by chronological age and competitive level into under-11 (U11, n = 130, boys = 75, girls = 55), under-13 (U13, n = 158, boys = 67, girls = 91), and under-15 (U15, n = 82, boys = 40, girls = 42) groups. This classification allowed for meaningful comparisons across different stages of growth and athletic development.

#### 2.1.1. Inclusion Criteria

Participants included healthy, registered youth handball players with at least two years of structured training at a club or regional center. They took part in organized conditioning, technical, and tactical sessions for at least 6 h per week.

#### 2.1.2. Exclusion Criteria

Participants with acute musculoskeletal injuries, recent illness or infection, cardiopulmonary conditions limiting maximal physical testing, or incomplete outcome data were excluded.

The study protocol was approved by the Ethics and Medical Research Committee of Farhat Hached University Hospital, Sousse, Tunisia (Opinion No. 24/2022; IRB No. 00,008,937 (OHRP); approval date: 13 January 2022). The study was conducted in accordance with the Declaration of Helsinki guidelines. All participants and their parents or legal guardians were informed of the study’s aims and procedures before participation. Written informed consent was obtained from the participants’ parents or legal guardians.

### 2.2. Experimental Procedures

All participants were assessed during the competitive period, between 5 February 2022 and 25 June 2022. Morphological and physiological measurements of each participant were conducted according to the specific parameters of categories.

Before data collection, testing was performed in the following sequence: (i) anthropometric and body composition assessments, (ii) flexibility assessment, (iii) jumping assessments, (iv) medicine ball throw, and (v) handgrip strength assessment. A 10 min rest interval was implemented between tests and trials to minimize fatigue. All testing sessions commenced at approximately 10 a.m.

Before testing, participants completed a 20 min standardized warm-up consisting of low-intensity jogging, dynamic mobility exercises, progressive running drills, and submaximal practice efforts relevant to the subsequent assessments.

Each participant was instructed and verbally encouraged to give his maximal effort. All participants were familiarized with the testing procedures to minimize learning effects.

All tests were conducted indoors.

### 2.3. Anthropometric Measurements

Weight was measured to the nearest 0.1 kg using a digital scale (Harpenden Balance Scale, Holtain Ltd., Crosswell, UK), and standing and sitting height were measured to the nearest 0.001 m using an appropriate stadiometer (Harpenden Portable Stadiometer, Holtain Ltd., Crosswell, UK). Body mass index (BMI) was calculated using the standard formula:

BMI = Weight/Height^2^.

Skin-fold thickness measurements were performed on the left side of the body at four sites: biceps, triceps, subscapular, and supra-iliac skin-folds. Measurements were taken in triplicate using a Harpenden Skinfold Caliper (Harpenden, Holtain Ltd., Crosswell, UK), and the mean values were used for subsequent analysis. The measurement error was 0.1 mm, and the reliability was 95%. Fat-free mass percentage (FFM%) was calculated as follows: FFM (%) = ((body mass − fat mass)/body mass) × 100 [29]. Arm span was measured in centimeters (cm) from the right to the left middle fingertip with the arms extended and abducted. Palm opening was measured in centimeters (cm) from the fingertip of the thumb to the fingertip of the little finger with all fingers abducted, and palm length from the mid-stylion to dactylion, measured in centimeters (cm).

### 2.4. Trunk Flexibility

Trunk flexibility was measured using the sit and reach test performed on a Wells bench (Banco de Wells Instant Flex Sanny, product code BW2002, Sanny, São Bernardo do Campo, São Paulo, Brazil), measured to the nearest centimeter (cm). Participants were instructed to stand with feet outstretched, with their knees extended, and to perform a maximal trunk flexion, reaching forward as far as possible. A 90° angle was maintained at the ankles, and the zero point was defined as the position where the fingertips just reached the toes. The distance between standing level and the fingertips was measured using a tape measure with negative values scored when reaching above standing level and positive values when reaching beyond.

### 2.5. Vertical Jump Tests

The vertical jump (VJ) was evaluated using both the squat jump (SJ) and the countermovement jump (CMJ). During the SJ, the participant was instructed to sink and to hold a squat position for 3 s. On the count of three, he was requested to jump as high as possible. A successful SJ trial was defined by the absence of any preparatory countermovement. For the CMJ, participant started from a standing position, rapidly descended to approximately 90° knee flexion, and immediately transitioned into maximal vertical jump. Verbal encouragements were constantly given to ensure high motivation. Each participant performed three trials for each jump. The best of three attempts was retained for analysis. The jumps were separated by a 2 min rest to ensure sufficient recovery. During vertical jump tests, participants were instructed to jump vertically for maximal height and to land in the same position and at the same place from takeoff to avoid lateral or horizontal displacement. To emphasize the use of leg extensors, participants were asked to maintain their torso in an upright position. Jumping performance was evaluated with the optical system Optojump Next (Microgate SRL, Bolzano, Italy). The apparatus comprises two bars placed 1 m apart and parallel to each other connected directly to a personal computer via a USB port.

### 2.6. Horizontal Jumping Tests

Each participant performed a series of horizontal jumps (HJ). The tests included a bilateral standing horizontal jump, and unilateral horizontal jumps with each of the two legs. Results were categorized into dominant and non-dominant leg for each player. For these tests, the participant stood stationary on both legs, with the toes aligned level with the start line, before jumping as far forward as possible. Participants were allowed the use of a countermovement with arms and body swing. The unilateral horizontal jumps required the athlete to begin by standing on the designated test leg with their toe touching the starting line and to jump as far forward as possible, landing on both feet. The distance jumped was measured from the take-off to the rearmost heel at landing using a metal tape measure, a 30 m metric steel tape measure (M1, Stanley, New Britain, CT, USA), and recorded in meters to the nearest centimeter. Participants were allowed three trials for each jump, with the longest distance jumped recorded for analysis. This test has been shown to be reliable with intraclass correlations between 0.80 and 0.95 [11].

### 2.7. Hand Explosive Power

Upper limb explosive power was measured using the medicine ball throw (MB) test. Participants were seated on the ground with their legs extended and feet apart. A 3 kg medicine ball was held with both hands at the neck. The ball was then thrown as far as possible by explosively extending the arms and forearms. The throwing distance, measured by tape measure (M1, 30 m; Stanley, New Britain, CT, USA) in centimeters from the throwing line to the ball’s landing point, was recorded. The best of three trials was retained.

The maximal isometric handgrip (HG) force was measured using a handheld handgrip dynamometer (T.K.K. 5401 Grip-D, Takei Scientific Instruments Co., Tokyo, Japan). Participants were familiarized with the dynamometer through three warm-up repetitions. The players performed two repetitions at maximum intensity with both the dominant and the non-dominant hand. Measurements were taken from a standing position, with the dynamometer aligned parallel to the body. In this position, the player was asked to exert maximal grip force without arm or wrist movement. For motivational purposes, the players were immediately informed of their performance. A rest period of three minutes elapsed between trials to minimize the effects of fatigue.

### 2.8. Statistical Analysis

All statistical analyses were performed using the “Statistical Package for Social Sciences” SPSS software version 23.0 (IBM Corp., Armonk, NY, USA). The normality of continuous variables was assessed using the Kolmogorov–Smirnov test. Homogeneity of variance was assessed using Levene’s test. As the retained variables did not show significant deviations from normality, data are presented as mean ± standard deviation (X ± SD) for all variables, and parametric statistical methods were utilized.

Anthropometric, body composition, and physical performance outcomes were analyzed separately using two-way analysis of variance (ANOVA), with sex, chronological age category, and sex × chronological age category interaction entered as fixed factors. Interaction effects were interpreted first. When a significant sex × chronological age category interaction was observed. Bonferroni-adjusted simple-effect comparisons were performed to identify the specific subgroup differences. When the interaction was not significant, the main effects of sex and chronological age category were interpreted, with Bonferroni-adjusted post-hoc comparisons applied for chronological age categories where appropriate. To avoid treating additional tests as independent primary analyses, one-way ANOVA and independent-samples *t*-tests were used only as simple-effect or post-hoc procedures when justified by the two-way ANOVA results.

Effect sizes for the ANOVA models were expressed as partial eta squared (η_p_^2^), interpreted as follows: values < 0.06 denote a low effect, values between 0.06 and 0.14 indicate a moderate effect, and values > 0.15 represent a large effect. Secondly, multiple linear regression analyses were conducted as exploratory cross-sectional associations between anthropometric characteristics and physical performance outcomes. Each physical performance variable was entered separately as a dependent variable, while age, BMI, FFM%, arm span, and palm opening were entered as independent anthropometric variables. Both full-model multiple linear regression and exploratory stepwise multiple linear regression were performed. The coefficients reported in the regression tables are unstandardized regression coefficients unless otherwise stated. Model-level information included the standard error of the estimate, R^2^, and overall model significance. The stepwise procedure was performed, with *p* < 0.05 for variable entry and *p* > 0.10 for variable removal.

For the multiple linear regression analyses, assumptions were examined by inspecting linearity, residual distribution, homoscedasticity, influential observations, and multicollinearity diagnostics. Multicollinearity of the variables was assessed by using tolerance and variance inflation factor (VIF) values. Tolerance values < 0.20 or VIF values > 5 were considered indicative of potentially problematic multicollinearity.

Regression analyses were presented separately from the main group-comparison analyses and were interpreted only as exploratory cross-sectional associations within the present sample. They were not interpreted as causal models or as evidence of future athletic performance. Statistical significance was set at *p* < 0.05.

Sex- and competitive age-category-specific percentile values were calculated for the retained anthropometric and physical performance variables at the 5th, 10th, 25th, 50th, 75th, 90th, and 95th percentiles. These percentiles were interpreted as descriptive reference distributions for comparable competitive Tunisian youth handball players.

## 3. Results

### 3.1. Anthropometric Characteristics and Body Composition

Descriptive statistics of anthropometric characteristics and body composition for all players according to sex and competitive age category are presented in Table 1. Data are expressed as mean ± standard deviation for boys and girls in the U11, U13, and U15 groups.

Chronological age differed significantly across the U11, U13, and U15 categories, as expected from the age-group classification (F(2364) = 1120.62, *p* < 0.001, η_p_^2^ = 0.860), whereas no significant sex effect (F(1364) = 0.68, *p* = 0.409, η_p_^2^ = 0.002) or sex × age category interaction (F(2364) = 0.40, *p* = 0.673, η_p_^2^ = 0.002) was observed for chronological age.

For BMI, a significant sex effect was observed (F(1364) = 20.61, *p* < 0.001, η_p_^2^ = 0.054), with girls showing higher BMI than boys in the U11 category. However, the age category effect (F(2364) = 2.59, *p* = 0.076, η_p_^2^ = 0.014) and the sex × age category interaction (F(2364) = 1.65, *p* = 0.194, η_p_^2^ = 0.009) were not significant.

Fat-free mass percentage showed a significant effect of sex (F(1364) = 118.33, *p* < 0.001, η_p_^2^ = 0.245), with boys presenting higher values than girls across chronological age categories.

This was accompanied by a non-significant difference between age categories (F(2364) = 1.02, *p* = 0.363, η_p_^2^ = 0.006), as well as a non-significant effect of sex × chronological age category interaction (F(2364) = 2.17, *p* = 0.115, η_p_^2^ = 0.012).

The arm span results revealed a significant effect for sex (F(1364) = 4.30, *p* = 0.039, η_p_^2^ = 0.012) and the chronological age category (F(2364) = 163.37, *p* < 0.001, η_p_^2^ = 0.473). It also revealed a significant sex × chronological age category interaction (F(2364) = 3.43, *p* = 0.033, η_p_^2^ = 0.019). The Bonferroni-adjusted comparisons revealed higher arm-span values in the older chronological age categories. However, the significant sex × chronological age category interaction indicated that the pattern of sex differences varied across categories.

The palm opening showed a significant sex effect (F(1364) = 19.45, *p* < 0.001, η_p_^2^ = 0.051) and chronological age category effect (F(2364) = 55.08, *p* < 0.001, η_p_^2^ = 0.232), as well as a significant sex × chronological age category interaction (F(2364) = 4.37, *p* = 0.013, η_p_^2^ = 0.023). The palm opening was greater in the older chronological age categories, and boys showed higher values than girls, particularly in the U15 category. However, the significant interaction indicates that the magnitude of the sex difference varied across chronological age categories.

### 3.2. Physical Performance Characteristics

Physical performance characteristics for all players, by sex and competitive age category, are presented in Table 2. The results are reported by distinguishing the main effects of sex, main effects of chronological age category, sex × chronological age category interactions, and Bonferroni-adjusted post-hoc comparisons. Data are expressed as mean ± standard deviation for boys and girls in the U11, U13, and U15 groups.

Flexibility showed a significant main effect of chronological age category (F(2364) = 4.49, *p* = 0.012, η_p_^2^ = 0.024), whereas the main effect of sex (F(1364) = 1.92, *p* = 0.167, η_p_^2^ = 0.005) and the sex × chronological age category interaction (F(2364) = 0.096, *p* = 0.909, η_p_^2^ = 0.001) were not significant. These results indicate that flexibility differed across chronological age categories, without evidence of a sex-specific pattern.

The squat jump showed a significant effect of sex (F(1364) = 39.80, *p* < 0.001, η_p_^2^ = 0.099) and chronological age category (F(2364) = 16.74, *p* < 0.001, η_p_^2^ = 0.084), while the sex × chronological age category interaction was not significant. Boys showed higher squat jump values than girls, and older chronological age categories generally showed higher values than younger categories. Likewise, the countermovement jump showed significant main effects of sex (F(1364) = 38.99, *p* < 0.001, η_p_^2^ = 0.097) and chronological age category (F(2364) = 23.12, *p* < 0.001, η_p_^2^ = 0.113), with no significant sex × chronological age category interaction. Boys showed higher countermovement jump values than girls, and older chronological age categories generally showed higher values than younger categories.

The 3 kg medicine ball throw showed significant main effects of sex (F(1364) = 92.42, *p* < 0.001, η_p_^2^ = 0.202) and chronological age category (F(2364) = 137.26, *p* < 0.001, η_p_^2^ = 0.430), whereas the sex × chronological age category interaction was not significant (F(2364) = 0.396, *p* = 0.673, η_p_^2^ = 0.002). Boys showed higher medicine ball throw values than girls, and older chronological age categories generally showed higher values than younger categories.

The mean 5 CMJ showed significant main effects of sex (F(1364) = 21.42, *p* < 0.001, η_p_^2^ = 0.056) and chronological age category (F(2364) = 22.34, *p* < 0.001, η_p_^2^ = 0.109), with no significant sex × chronological age category interaction. The best 5 CMJ also showed significant effects of sex (F(1364) = 27.61, *p* < 0.001, η_p_^2^ = 0.071) and chronological age category (F(2364) = 20.91, *p* < 0.001, η_p_^2^ = 0.103), with no significant interaction (F(2364) = 0.988, *p* = 0.373, η_p_^2^ = 0.005). For both variables, boys showed higher values than girls, and older chronological age categories generally showed higher values than younger categories.

The bilateral horizontal jump showed significant main effects of sex (F(1364) = 59.66, *p* < 0.001, η_p_^2^ = 0.141) and chronological age category (F(2364) = 52.76, *p* < 0.001, η_p_^2^ = 0.225), as well as a significant sex × chronological age category interaction (F(2364) = 3.622, *p* = 0.028, η_p_^2^ = 0.020). These results indicate that boys generally showed higher horizontal-jump values than girls, but the magnitude of the sex difference varied according to chronological age category.

The dominant-leg horizontal jump showed significant main effects of sex (F(1364) = 39.48, *p* < 0.001, η_p_^2^ = 0.098) and chronological age category (F(2364) = 58.31, *p* < 0.001, η_p_^2^ = 0.243), together with a significant sex × chronological age category interaction (F(2364) = 4.198, *p* = 0.016, η_p_^2^ = 0.023). The non-dominant-leg horizontal jump also showed significant main effects of sex (F(1364) = 38.22, *p* < 0.001, η_p_^2^ = 0.095) and chronological age category (F(2364) = 48.29, *p* < 0.001, η_p_^2^ = 0.210), with a significant interaction (F(2364) = 6.826, *p* = 0.001, η_p_^2^ = 0.036). These findings indicate that unilateral horizontal jump performance differed by sex and chronological age category, with the sex difference varying across age categories.

The dominant handgrip strength showed significant main effects of sex (F(1364) = 28.63, *p* < 0.001, η_p_^2^ = 0.073) and chronological age category (F(2364) = 99.30, *p* < 0.001, η_p_^2^ = 0.353), as well as a significant sex × chronological age category interaction (F(2364) = 6.393, *p* = 0.002, η_p_^2^ = 0.034). The non-dominant handgrip strength showed similar results, with significant main effects of sex (F(1364) = 18.35, *p* < 0.001, η_p_^2^ = 0.048) and chronological age category (F(2364) = 81.36, *p* < 0.001, η_p_^2^ = 0.309), and a significant interaction (F(2364) = 5.763, *p* = 0.003, η_p_^2^ = 0.031). Boys generally showed higher handgrip strength values than girls, particularly in the older chronological age categories.

### 3.3. Multiple Regression Equations

#### The Full-Model Multiple Regression

Both full-model and exploratory stepwise multiple linear regression analyses were used to examine cross-sectional associations between anthropometric characteristics and physical performance outcomes. Each physical performance variable was entered separately as a dependent variable, while age, BMI, FFM%, arm span, and palm opening were entered as independent variables. The results of full-model regression of anthropometric characteristics and physical performance analysis are presented in Table 3.

### 3.4. Exploratory Stepwise Multiple Linear Regression Models

The results of the exploratory stepwise multiple linear regression analyses are presented in Table 4. Each physical performance outcome was entered separately as a dependent variable, while age, BMI, FFM%, arm span, and palm opening were treated as independent anthropometric variables. The coefficients reported in the table are unstandardized regression coefficients.

### 3.5. Percentiles

The percentile values were calculated to estimate the 5th, 10th, 25th, 50th, 75th, 90th, and 95th percentiles of boys and girls by age category. The presented percentiles values are useful to describe the distribution of anthropometric and physical performance scores within the present sample of competitive Tunisian youth handball players (Table 5). They may support descriptive benchmarking and training monitoring among comparable players of the same sex and chronological age category.

## 4. Discussion

The present cross-sectional study examined the anthropometric, body composition and physical performance characteristics in competitive Tunisian youth handball players according to sex and chronological age category. By assessing large and diverse regional training centers and clubs, this research provides updated sex- and competitive-age-category-specific reference values for youth Tunisian handball players, a population that remains underrepresented in the literature despite its growing participation in organized handball.

The main findings indicate that several anthropometric and physical performance variables differed across chronological age categories and between sexes. In general, older age categories showed higher values on strength- and power-related outcomes, while boys outperformed girls on several explosive and strength assessments. Flexibility exhibited a less marked sex-related pattern. These results align with the multidimensional nature of youth handball performance, which is influenced by body size, body composition, neuromuscular capacity, training exposure, and sport-specific motor development [1,2,3,4,5,6,7,8].

The field tests used in our study are functionally relevant to handball. Match-play and performance studies show that handball requires repeated high-intensity actions, including jumps, throws, accelerations, decelerations, and physical duels [1,2,3,4,5]. Lower-limb explosive performance is important for jumping, acceleration, and horizontal propulsion, whereas upper-limb power and grip strength are relevant for throwing, ball control, and contact situations [9,10,11,12,13,14,15]. Therefore, the assessment battery used in this study provides practical information about physical capacities related to the demands of youth handball.

In the youngest chronological age categories, sex-related differences were less pronounced for several variables. This observation may be attributed to the inclusion of participants in prepubertal or early pubertal stages, during which boys and girls typically exhibit similar anthropometric and neuromuscular characteristics [19,21]. However, biological maturation was not directly assessed in the present study; therefore, this explanation should be considered plausible but unmeasured. During adolescence, previous studies have shown that sex-related differences in body composition, strength, and power may become more evident, partly because of differences in the timing and tempo of maturation [19,21,24]. In the present sample, higher values observed in boys in older competitive age categories may therefore be consistent with maturation-related patterns reported in the literature, but they cannot be attributed directly to pubertal or hormonal mechanisms.

The anthropometric and body composition results highlight that body mass index (BMI) has a limited utility as a performance indicator in youth athletes. While girls showed higher BMI values than boys in the youngest age category, no significant sex differences were observed at older ages, and BMI stayed about the same across age groups for both boys and girls. This finding is in accordance with previous evidence indicating that BMI lacks sensitivity in the athletic population because it does not distinguish between fat and lean mass [12,26]. In contrast, fat-free mass percentage differed between sexes, with higher values observed in boys. This finding is consistent with previous literature indicating that body composition characteristics are associated with strength- and power-related performance in youth athletes [19,24,30]. However, as biological maturation and hormonal status were not assessed, the present data cannot determine whether these differences are attributable to pubertal or hormonal mechanisms.

Anthropometric dimensions directly relevant to handball performance, particularly arm span and palm opening, differed across chronological age categories. These characteristics are frequently reported as advantageous in handball, as they facilitate ball control, passing accuracy, shooting mechanics, and spatial dominance during both offensive and defensive actions [1,13]. The progressive development of these traits underscores the need to interpret anthropometric advantages in youth athletes cautiously, as early superiority may partly reflect unmeasured maturational differences rather than long-term performance potential.

Lower-limb explosive performance, assessed using squat jump, countermovement jump, and horizontal jump tests, differed according to sex and competitive age category. Older competitive age categories generally showed higher values than younger categories, and boys showed higher values than girls in several jump outcomes. These differences are relevant because jumping and horizontal propulsion are important components of handball-specific actions. Previous findings in youth handball players and other team sports players indicate that jump performance is sensitive to changes in muscle mass, tendon properties, and neuromuscular coordination, and can effectively distinguish between different performance levels [31,32,33]. Given the importance of jumping actions in handball, these findings have clear practical relevance for training and performance monitoring.

Upper-limb explosive power, evaluated using the 3 kg medicine ball throw, also differed according to sex and competitive age category, with higher values observed in older categories and in boys. Medicine ball throwing tasks closely replicate handball-specific throwing actions and are correlated with throwing velocity and overall performance in youth athletes [14,15]. Similarly, handgrip strength varied across competitive age categories and showed sex differences, especially in older groups. Handgrip strength is relevant for ball-handling and throwing efficiency. However, these findings should be interpreted with caution because they may reflect combined influences of body size, training experience, neuromuscular factors, and unmeasured biological maturation, rather a single explanatory mechanism [14,31].

The percentile reference values found in our study represent a key practical contribution. They provide sex- and age-specific reference distributions to help coaches, trainers, and sport scientists compare individual players with their peers from similar competitive and cultural backgrounds. These values support benchmarking, training monitoring, and the identification of relative strengths and weaknesses.

The regression analyses were interpreted with caution because they examined cross-sectional associations between anthropometric characteristics and physical performance outcomes. Such models cannot establish causal relationships or determine whether anthropometric characteristics predict future athletic performance. In youth athletes, anthropometric variables such as body size, fat-free mass, arm span, and hand dimensions may be interrelated because of growth and maturation, which can affect the interpretation of individual regression coefficients [19,20,24]. Therefore, these findings should be considered exploratory and complementary to the main objective of establishing sex- and competitive-age-category-specific reference values.

This study demonstrates several strengths. It included a relatively large overall sample of competitive youth handball players and provided sex- and competitive-age-category-specific values across a broad set of anthropometric and physical performance variables. It also focused on a North African youth handball population for which reference data remain limited [28]. Nevertheless, the study has some limitations. First, the cross-sectional design prevents conclusions about individual development and training effects. Second, biological maturation was not assessed using maturity offset, peak height velocity, Tanner stage, or skeletal age; therefore, the results cannot be interpreted as maturity-adjusted reference values. Third, no formal a priori sample-size calculation was performed; some sex-by-age subgroups were relatively small, particularly in the U15 category.

In practical terms, the findings support the use of multidimensional assessment in youth handball players. Coaches and trainers should consider anthropometric characteristics, body composition, jumping ability, throwing performance, handgrip strength, and flexibility together rather than relying on a single test. The reference percentile values provided here may assist in monitoring players within comparable age and sex categories, but they should be used alongside technical, tactical, psychological, training-load, and maturation-related information. Future longitudinal research should incorporate direct or validated indirect indicators of biological maturation, training-load monitoring, and repeated performance assessments to determine whether early physical advantages persist over time and how they relate to long-term player development [19,20,25,27].

## 5. Conclusions

This study provides new evidence on sex- and competitive age-category-specific anthropometric and physical performance reference values of Tunisian youth handball players aged 9 to 15. Our findings indicate that the group of older competitive age categories generally showed higher strength and power values, while boys outperformed girls on selected jumping, throwing, and handgrip strength measures. These results may establish regional benchmarks for Tunisian youth handball and help coaches, physical trainers, and sport scientists interpret the physical profiles of young handball players within a comparable competitive context.

## Figures and Tables

**Table 1:** Descriptive statistics of anthropometric and body composition variables in handball players by competitive age category and sex.

Variables					Sex			Age			Sex × Age		
		U11	U 13	U15	F(1364)	*p* Value	η_p_^2^	F(2364)	*p* Value	η_p_^2^	F(2364)	*p* Value	η_p_^2^
Age (years)	Boys	10 ± 0.5	12 ± 0.6 b	13.8 ± 0.5 b	0.68	0.409	0.002	1120.62	<0.001	0.860	0.40	0.673	0.002
	Girls	10.2 ± 0.5	12 ± 0.6 b	13.5 ± 0.5 b									
BMI (kg/m^2^)	Boys	17.3 ± 2.6	17.8 ± 3	18.7 ± 3	20.61	<0.001	0.054	2.59	0.076	0.014	1.65	0.194	0.009
	Girls	19.8 ± 4.1 a	18.9 ± 3.9	20 ± 3.3									
FFM%	Boys	66.5 ± 4.9	65.9 ± 4.5	65.8 ± 5.4	118.33	<0.001	0.245	1.02	0.363	0.006	2.17	0.115	0.012
	Girls	58.8 ± 5.8 a	60.8 ± 6.3 a	59 ± 6.1 a									
Arm span (cm)	Boys	146.3 ± 8.3	156.4 ± 9.7 b	170.7 ± 12.2 b	4.30	0.039	0.012	163.37	<0.001	0.473	3.43	0.033	0.019
	Girls	145 ± 7.3	157.2 ± 8 b	165.2 ± 8 b									
Palm opening (cm)	Boys	19.1 ± 1.2	20.3 ± 1.1 b	21.5 ± 1.7 b	19.45	<0.001	0.051	55.08	<0.001	0.232	4.37	0.013	0.023
	Girls	18.9 ± 1.1	19.9 ± 1.2 b	20.3 ± 1.5 a									

Values are presented as mean ± SD; U11: under 11 years; U13: under 13 years; U15: under 15 years; a: significantly different from boys at *p* < 0.05; b: significantly different from previous age at *p* < 0.05; FFM%: fat-free mass percentage; BMI: body mass index.

**Table 2:** Physical performance variables in handball players by competitive age category and sex.

Variables					Sex			Age			Sex × Age		
		U11	U13	U15	F(1364)	*p* Value	η_p_^2^	F(2364)	*p* Value	η_p_^2^	F(2364)	*p* Value	η_p_^2^
Flexibility (cm)	Boys	−0.98 ± 6.07	0.76 ± 6.62 b	1.63 ± 5.34 b	1.92	0.167	0.005	4.49	0.012	0.024	0.096	0.909	0.001
	Girls	0.21 ± 5.32	1.73 ± 5.48 b	2.1 ± 5.66 b									
SJ (cm)	Boys	17.6 ± 3.3	19.7 ± 4.1 b	20.5 ± 3.3 b	39.80	<0.001	0.099	16.74	<0.001	0.084	0.544	0.581	0.003
	Girls	15.4 ± 3.4 a	16.7 ± 3.9 ab	18.2 ± 3.6 ab									
CMJ (cm)	Boys	17.8 ± 3.3	20.9 ± 4.1 b	21.5 ± 3.5 b	38.99	<0.001	0.097	23.12	<0.001	0.113	2.041	0.131	0.011
	Girls	15.9 ± 3.9 a	17.3 ± 4.2 ab	19.3 ± 4 ab									
MB throw 3 kg (m)	Boys	1.97 ± 0.4	2.52 ± 0.46 b	3.1 ± 0.62 b	92.42	<0.001	0.202	137.26	<0.001	0.430	0.396	0.673	0.002
	Girls	1.52 ± 0.32 a	1.97 ± 0.47 ab	2.61 ± 0.63 ab									
Mean 5 CMJ (cm)	Boys	16 ± 3.2	18.5 ± 3.7 b	20 ± 3.6	21.42	<0.001	0.056	22.34	<0.001	0.109	1.473	0.231	0.008
	Girls	15.1 ± 3.3	16.1 ± 3.9 a	17.8 ± 3.7									
Best 5 CMJ (cm)	Boys	17.7 ± 3.5	20.1 ± 4.1 b	21.4 ± 3.5	27.61	<0.001	0.071	20.91	<0.001	0.103	0.988	0.373	0.005
	Girls	16.2 ± 3.4	17.3 ± 4 a	19.2 ± 3.7									
Horizontal jump (m)	Boys	1.33 ± 0.19	1.46 ± 0.23 b	1.54 ± 0.2	59.66	<0.001	0.141	52.76	<0.001	0.225	3.622	0.028	0.020
	Girls	1.09 ± 0.18 a	1.32 ± 0.19 ab	1.43 ± 0.19 b									
Horizontal jump dominant (m)	Boys	1.2 ± 0.2	1.3 ± 0.2 b	1.4 ± 0.2	39.48	<0.001	0.098	58.31	<0.001	0.243	4.198	0.016	0.023
	Girls	1 ± 0.2 a	1.2 ± 0.2 ab	1.4 ± 0.2 b									
Horizontal jump non-dominant (m)	Boys	1.22 ± 0.21	1.32 ± 0.22	1.4 ± 0.26	38.22	<0.001	0.095	48.29	<0.001	0.210	6.826	0.001	0.036
	Girls	0.97 ± 0.17 a	1.2 ± 0.19 ab	1.35 ± 0.2 b									
HG dominant (kg)	Boys	16.9 ± 4	21.8 ± 6.1 b	30.2 ± 6.6 b	28.63	<0.001	0.073	99.30	<0.001	0.353	6.393	0.002	0.034
	Girls	15.8 ± 3.5	19.8 ± 6 b	23.9 ± 5.1 ab									
HG non-dominant (kg)	Boys	15.4 ± 3.8	19.3 ± 5.5 b	27 ± 6.8 b	18.35	<0.001	0.048	81.36	<0.001	0.309	5.763	0.003	0.031
	Girls	14.9 ± 3.7	18 ± 5.6 b	21.7 ± 5.2 ab									

Values are presented as mean ± SD; U11: under 11 years; U13: under 13 years; U15: under 15 years; a: significantly different from boys at *p* < 0.05; b: significantly different from previous age at *p* < 0.05; SJ: squat jump; CMJ: countermovement jump; MB throw 3 kg: medicine ball throw; HG: handgrip.

**Table 3:** Full-model regression of anthropometric characteristics and physical performance.

Variables	Coefficients						Standard Error of the Estimate	R^2^	*p* Value
	Constant	Age	BMI	FFM%	Arm Span	Palm Opening			
Flexibility (cm)	−222.251	0.729	0.615	−0.140	−0.171	1.643	5.796	0.157	<0.001
SJ (cm)	−28.518	0.758	−0.299	0.052	0.035	0.266	3.314	0.276	<0.001
CMJ (cm)	−82.951	0.907	−0.293	0.031	0.020	0.409	3.366	0.321	<0.001
MB throw 3 kg (m)	−9.698	0.154	0.034	−0.007	0.023	−0.016	0.380	0.669	<0.001
Mean 5 CMJ (cm)	−26.113	1.080	−0.229	0.066	0.039	0.372	3.050	0.393	<0.001
Best 5 CMJ (cm)	−41.939	0.982	−0.241	0.057	0.028	0.497	3.342	0.342	<0.001
Horizontal jump (m)	1.919	0.041	−0.008	0.003	0.004	0.200	0.194	0.276	<0.001
Horizontal jump dominant (m)	−1.364	0.035	−0.002	0.001	0.004	0.028	0.182	0.279	<0.001
Horizontal jump non-dominant (m)	−1.012	0.029	−0.006	0.001	0.007	0.011	0.204	0.255	<0.001
HG dominant (kg)	−33.070	1.650	0.142	−0.194	0.234	0.274	4.332	0.672	<0.001
HG non-dominant (kg)	−42.853	1.309	0.297	−0.173	0.252	−0.118	4.278	0.617	<0.001

Function: variables = constant + (age coefficient × age) + (BMI coefficient × BMI) + (FFM% coefficient × FFM%) + (arm span coefficient × arm span) + (palm opening coefficient × palm opening); SJ: squat jump; CMJ: countermovement jump; MB throw 3 kg: medicine ball throw; HG: handgrip. Coefficients are unstandardized regression coefficients. SEE: standard error of the estimate; R^2^: coefficient of determination.

**Table 4:** Stepwise multiple linear regression models of anthropometric variables and physical performance outcomes.

Variables	Coefficients						Standard Error of the Estimate	R^2^	*p* Value
	Constant	Age	BMI	FFM%	Arm Span	Palm Opening			
Flexibility (cm)	−259.136	-	0.599	-	−0.128	1.65	5.8139	0.137	<0.001
SJ (cm)	17.457	0.954	−0.535	-	-	-	3.324	0.251	<0.001
CMJ (cm)	15.59	1.177	−0.533	-	-	-	3.398	0.288	<0.001
MB throw 3 kg (m)	−1.694	0.157	-	−0.016	0.022	-	0.38087	0.660	<0.001
Mean 5 CMJ (cm)	7.865	1.221	−0.336	-	-	0.525	3.04309	0.385	<0.001
Best 5 CMJ (cm)	15.477	1.192	−0.558	-	-	-	3.4046	0.298	<0.001
Horizontal jump (m)	0.666	0.041	-	-	0.007	-	0.19367	0.263	<0.001
Horizontal jump dominant (m)	0.367	0.041	−0.017	-	-	0.037	0.18333	0.255	<0.001
Horizontal jump non-dominant (m)	0.52	0.031	-	-	0.008	-	0.2034	0.246	<0.001
HG dominant (kg)	−24.042	1.619	-	−0.22	0.267	-	4.3004	0.670	<0.001
HG non-dominant (kg)	−18.582	1.28	-	−0.221	0.243	-	4.2593	0.611	<0.001

Function: variables = constant + (age coefficient × age) + (BMI coefficient × BMI) + (FFM% coefficient × FFM%) + (arm span coefficient × arm span) + (palm opening coefficient × palm opening); SJ: squat jump; CMJ: countermovement jump; MB throw 3 kg: medicine ball throw; HG: handgrip. Coefficients are unstandardized regression coefficients. SEE: standard error of the estimate; R^2^: coefficient of determination.

**Table 5:** Specific percentile reference values for physical performance according to competitive age category and sex.

Variables		Boys							Girls						
		5%	10%	25%	50%	75%	90%	95%	5%	10%	25%	50%	75%	90%	95%
Flexibility (cm)	U11	−10.5	−9.3	−5.6	−0.3	3.4	6.5	8.2	−8.3	−7.6	−3.0	0.7	3.3	7.5	8.4
	U13	−9.5	−8.2	−3.8	3.1	5.1	8.7	10.9	−7.9	−4.8	−1.2	1.7	4.3	9.4	10.9
	U15	−7.2	−6.8	−0.6	1.8	5.6	8.0	8.8	−6.7	−5.3	−1.4	3.1	6.1	7.3	9.4
SJ (cm)	U11	12.2	13.7	15.2	17.8	19.8	21.7	22.3	10.2	11.3	12.6	15.4	17.4	19.7	21.6
	U13	13.6	14.3	16.6	20.2	22.9	24.8	25.7	11.9	12.6	13.8	16.3	19.0	22.0	24.5
	U15	15.7	16.8	18.4	20.2	23.4	24.6	24.6	13.3	13.8	15.3	18.0	20.9	23.3	24.8
CMJ (cm)	U11	12.4	13.1	16.0	18.1	19.2	22.2	23.3	10.8	11.5	12.8	15.6	18.2	21.5	23.2
	U13	14.1	15.5	18.7	21.4	23.0	25.2	27.6	12.0	12.3	14.1	16.6	20.0	23.7	24.4
	U15	15.8	16.7	19.1	21.6	24.4	25.8	26.4	13.8	14.4	16.1	18.9	22.3	25.2	26.3
MB throw 3 kg (m)	U11	1.36	1.43	1.66	1.90	2.32	2.48	2.62	0.99	1.11	1.35	1.52	1.67	1.81	2.15
	U13	1.63	1.83	2.30	2.53	2.79	3.08	3.21	1.12	1.41	1.63	2.01	2.27	2.63	2.66
	U15	2.30	2.34	2.58	3.18	3.53	3.82	3.94	1.54	1.75	2.21	2.73	2.99	3.21	3.34
Mean 5 CMJ (cm)	U11	9.9	11.3	13.6	16.4	18.3	19.9	20.9	10.9	11.4	12.5	15.2	16.8	19.6	20.6
	U13	12.4	14.0	15.8	18.4	20.6	22.5	25.3	10.5	11.7	13.2	15.3	18.4	21.3	23.7
	U15	15.1	15.6	17.0	19.3	22.7	24.8	25.4	13.7	14.1	14.9	17.3	20.1	22.9	23.8
Best 5 CMJ (cm)	U11	11.2	11.9	15.5	18.1	20.3	21.7	22.6	11.9	12.3	13.1	16.0	18.3	20.7	21.9
	U13	13.4	15.1	17.2	20.1	22.8	24.9	27.2	12.0	12.9	14.6	16.8	19.3	22.8	25.1
	U15	16.3	16.6	19.2	21.0	24.2	25.5	26.2	14.3	15.4	16.5	18.5	22.0	24.7	25.6
Horizontal jump (m)	U11	1.04	1.10	1.20	1.35	1.45	1.58	1.63	0.80	0.83	0.98	1.07	1.23	1.29	1.36
	U13	1.12	1.16	1.30	1.45	1.64	1.75	1.80	1.03	1.08	1.19	1.32	1.45	1.54	1.65
	U15	1.26	1.27	1.40	1.52	1.63	1.81	1.87	1.11	1.18	1.31	1.44	1.56	1.65	1.72
Horizontal jump dominant (m)	U11	1.01	1.03	1.08	1.19	1.30	1.42	1.47	0.68	0.75	0.88	0.96	1.10	1.23	1.33
	U13	1.01	1.07	1.13	1.34	1.45	1.55	1.60	0.89	0.92	1.05	1.13	1.32	1.44	1.53
	U15	1.05	1.15	1.27	1.39	1.48	1.75	1.81	1.08	1.11	1.21	1.32	1.47	1.61	1.78
Horizontal jump non-dominant (m)	U11	0.88	0.96	1.10	1.23	1.35	1.47	1.57	0.71	0.78	0.87	0.98	1.10	1.21	1.23
	U13	1.00	1.03	1.13	1.32	1.52	1.60	1.63	0.92	0.98	1.08	1.19	1.33	1.46	1.49
	U15	1.09	1.09	1.21	1.34	1.54	1.74	1.84	1.04	1.09	1.19	1.35	1.48	1.62	1.71
HG dominant (kg)	U11	12.0	12.6	13.9	16.2	19.2	22.3	23.5	10.2	11.7	14.1	15.7	17.9	20.2	21.5
	U13	13.4	15.3	17.9	20.9	24.0	31.3	33.7	11.9	12.7	15.6	19.2	23.2	26.6	30.0
	U15	19.9	22.5	25.3	31.1	34.5	39.6	40.0	16.7	18.2	21.0	23.2	26.4	28.7	34.3
HG non-dominant (kg)	U11	10.7	11.6	12.4	14.9	17.3	19.8	22.0	9.1	10.8	13.2	14.9	16.3	19.7	20.7
	U13	12.0	13.0	15.8	18.3	21.4	28.2	29.3	10.5	11.8	13.7	17.4	20.9	24.6	26.9
	U15	17.2	18.1	20.9	27.8	32.6	36.8	37.5	15.4	15.7	17.7	20.9	23.9	28.2	32.0

U11: under 11 years; U13: under 13 years; U15: under 15 years; SJ: squat jump; CMJ: countermovement jump; MB throw 3 kg: medicine ball throw; HG: handgrip.

## Data Availability

The original contributions presented in this study are included in the article. Further inquiries can be directed to the corresponding author.
